# Identification of Volatiles Produced by *Cladosporium cladosporioides* CL-1, a Fungal Biocontrol Agent That Promotes Plant Growth

**DOI:** 10.3390/s131013969

**Published:** 2013-10-16

**Authors:** Diby Paul, Kyung Seok Park

**Affiliations:** Microbial Plant Activation Lab, Agricultural Microbiology Division, National Academy of Agricultural Science, RDA, Suwon 441-707, Korea

**Keywords:** volatile organic compounds (VOCs), *Cladosporium cladosporioides*, plant growth promotion, tobacco, SPME-GC-MS

## Abstract

Certain microbial Volatile Organic Compounds (VOCs) have been reported to enhance the growth and development of plants. The biocontrol fungi, *Cladosporium cladosporioides* CL-1 significantly improved the growth of tobacco seedlings *in vitro* when they were co-cultivated without physical contact. SPME Quadrupole GC/MS/MS revealed that CL-1 emited the volatiles α-pinene, (−)-trans-caryophyllene, tetrahydro-2,2,5,5-tetramethylfuran, dehydroaromadendrene, and (+)-sativene. Potential roles of these volatiles in plant growth and development are discussed. Even though there were several fungal VOCs reported in the past that could influence plant growth, their exact mechanisms of action are not fully known. Fungal VOC-mediated plant growth promotion requires in-depth study in order for this technology to be used in large scale for crops, especially those grown under greenhouse conditions.

## 1. Introduction

In the rhizosphere, many microorganisms including bacteria and fungi are abundantly present, most often organized in micro-colonies, and grow by consuming various nutrients secreted by the plant roots. Some of these microorganisms benefit the plant both directly and indirectly, leading to the stimulation of plant growth. Apart from inducing growth promotion, these beneficial microbes suppress disease-causing microbes and accelerate nutrient availability and assimilation. Thus, in the quest to improve soil fertility and crop yield and to reduce the negative impacts of chemical fertilizers on the environment, there is a need to exploit these microbes for continued beneficial agricultural purposes. Beneficial microbes have been applied to a wide range of agricultural species for the purpose of growth enhancement, including increased seed emergence, plant weight, crop yield and disease control [1–4]. Several reports confirm the efficacy of this technology and their suitability as components in sustainable agricultural practices.

Research on Plant Growth Promoting Rhizobacteria (PGPR) and Plant Growth Promoting Fungi (PGPF) has been increasing at a fast pace since the term PGPR was first used by Kloepper and coworkers in the late 1970s [1]. The various mechanisms of action of these microbes in bringing beneficial effects of growth promotion, disease suppression, induced systemic resistance (ISR), *etc.*, to the plants have been reported. Beneficial microbes enhance the nutrient status of host plants by biological N_2_ fixation, increasing the availability of nutrients in the rhizosphere, increasing root surface area, and various combinations of these activities [5]. The vast majority of studies that investigate the mode of action of these plant growth promoting microorganisms have focused on their influences on plant growth regulating substances [2,3,6,7].

Fungi produce various mixtures of gas-phase, carbon-based compounds called volatile organic compounds (VOCs) that due to their small size are able to diffuse through the atmosphere and soils. Several fungal VOCs have been identified to occur as either simple hydrocarbons, heterocycles, aldehydes, ketones, alcohols, phenols, thioalcohols, thioesters, benzene derivatives or cyclohexanes [8,9]. Ryu *et al.* [10] first reported that the microbial VOCs 2,3-butanediol and acetoin promoted the growth of *Arabidopsis thaliana.* PGPF-derived VOC mediated growth promotion in plants was first reported by Yamagiwa *et al.* [11]. VOCs of certain PGPFs including the biocontrol fungus *Trichoderma* have been shown to enhance plant growth [7,11]. Here, we report the plant growth promoting potential of the PGPF *Cladosporium cladosporioides* CL-1. This isolate was originally obtained from the rhizosphere of red pepper and has been proven earlier to induce growth promotion in paprika, tobacco and *A. thaliana.* Some of volatiles produced by CL-1 were identified in this study using solid phase microextraction (SPME) coupled to gas chromatography–mass spectrometry (GC-MS).

## 2. Experimental Section

### 2.1. Storage and Growth of Microbial Cultures and Tobacco Seedlings Used

*Cladosporium cladosporioides* CL-1 was stored in Potato Dextrose Agar (PDA) medium at 4 °C and subcultured onto fresh PDA for the assays. For the purpose of volatile studies, CL-1 was grown on PDA at 28 °C for 7 days. Seeds of tobacco cv. *Xanthi* were stored at 5 °C and grown on Murashige and Skoog (MS) medium. All chemicals used in the study, unless otherwise stated, were obtained from Sigma (Korea).

### 2.2. Evaluation of Tobacco Growth Promotion by Volatiles Produced by CL-1

**Figure 1.**
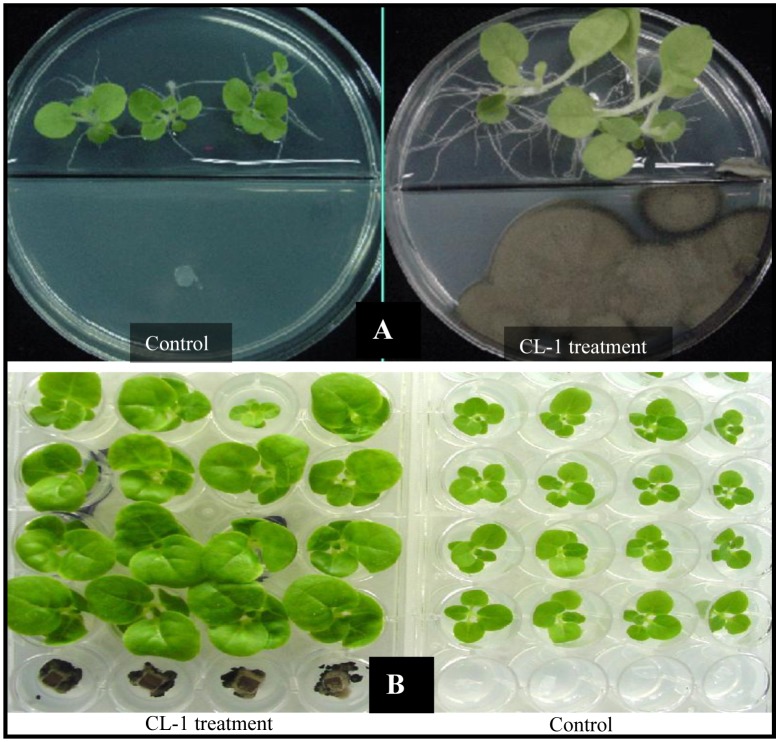
Tobacco growth promotion by volatiles produced by *Cladosporium cladosporioides* CL-1. (**A**) The I plate was used to co-culture tobacco seedlings and *C. cladosporioides* culture without physical contact; (**B**) 24-well culture plates were used to test the growth promoting effect of volatiles of *C. cladosporioides*.

Tobacco seeds were surface sterilized via 2 min washing in sterile water, followed by 10 min soaking in 75% ethanol and 10 min immersion in 3% (vol/vol) sodium hypochlorite. The treated seeds were rinsed four times with sterile distilled water. One seed per well was placed on MS agar (MSA; 0.8%) amended with 3% sucrose in 24-well plates (Falcon, NJ, USA) with each well containing 1 mL of the medium. Four wells at one end of the plate were reserved for culturing CL-1; each well was filled with 1 mL PDA and was inoculated with a 5 mm diameter plug of CL-1 culture (agar plugs taken from 7 days-old CL-1 culture on PDA). Plates were prepared in triplicate and a control plate (without CL-1) was also maintained. The plates were sealed and aseptically maintained in a growth chamber set at 25 °C with a 14 h light and 10 h dark regime for 4 weeks (Figure 1B). Fresh weights of seedlings were collected and the data analyzed.

The experiment was repeated using I-plates (Figure 1A) with one side containing 10 mL MSA and the other side containing 10 mL PDA. Tobacco seeds were placed on MSA and CL-1 was inoculated at the PDA side. These plates were sealed and placed in a growth chamber under the conditions described above for 4 weeks. The growth of seedlings and their root proliferation were visually compared with those in control plates to confirm growth promotion as in 24-well plates.

### 2.3. Identification of Volatiles Produced by CL-1 Using SPME-GC-MS

Twenty mL glass vials were used for capturing the volatiles produced by CL-1. After inoculation with a 5 mm disc of fresh culture, each vial was closed with a sterile cotton plug and incubated for 5 days. After purging the headspace with sterile synthetic air, the vial was sealed gas tight. The vial was further incubated for 6 h before sampling of volatiles. The volatiles were collected using solid phase microextraction (SPME) fiber assemblies (Supelco, Bellefonte, PA, USA) for 30 min at 25 °C. The fiber was coated with 65 μm polydimethylsiloxane (PDMS)/divinylbenzene (DVB). Following the sampling, the SPME fiber was immediately inserted into the GC injector, and the volatiles bound to the fiber were thermally desorbed for 3 min at 250 °C. A Varian VF-5MS (5% diphenyl-95% polymethylsiloxane) capillary column (30 m × 0.25 mm × 0.25 μm) (Varian, Palo Alto, CA, USA) was used for volatile separation. Helium was used as the carrier gas at a flow rate of 1 mL·min^−1^. The oven temperature program consisted of 40 °C (hold 2 min), 10 °C/min to 200 °C, and 25 °C/min to 260 °C (hold 5 min). The quadrupole mass detector was operated at 150 °C in the electron impact ionisation (EI) at 70 eV. The ion source temperature was set at 230 °C, and the transfer line was set at 280 °C. The mass acquisition range was 40–400 *m*/*z*.

As the aim of the current work was to prove the growth promotion potential of volatiles of CL-1 in plants and also to identify them, SPME-GC-MS analysis focused on the qualitative analysis of the volatiles. Individual peaks were identified on the basis of their fragmentation patterns and identities were confirmed by comparing the mass spectra with the data system libraries (Wiley and NIST-2007).

## 3. Results and Discussion

**Figure 2.**
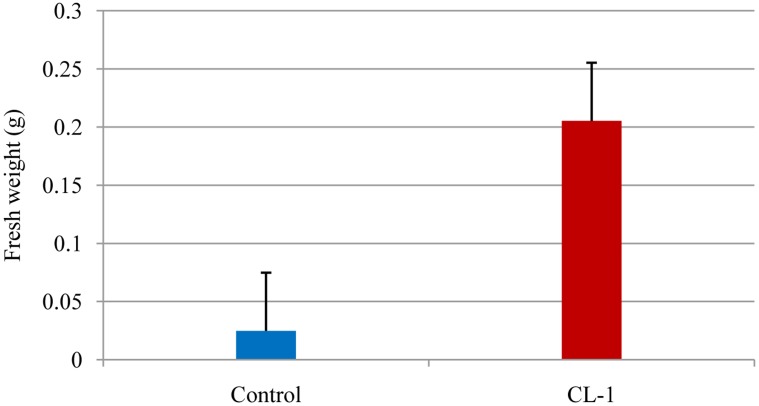
Tobacco seedling growth promotion by volatiles of CL-1. Volatiles of CL-1 significantly enhanced growth of tobacco seedlings in terms of fresh weight.

Plant growth-promoting rhizobacteria (PGPR) have been applied to a wide range of agricultural crops for the purpose of growth enhancement, including increased seed germination, plant weight, and yield [12,13]. A number of mechanisms have been suggested for rhizobacteria-mediated plant growth promotion: (i) increased phosphate solubilization [14]; (ii) secretion of plant growth-promoting substances such as auxins and cytokinins [15,16] and (iii) production of antibiotics and hydrogen cyanide, which indirectly promote plant growth by inhibiting the growth of deleterious microorganisms in the rhizosphere [17]. Our results showed that the fungal plant growth promoting agent CL-1 significantly improved the growth of tobacco seedlings and their root development through the production of VOCs (Figures 1 and 2). Volatiles could be an effective tool for rhizosphere microorganisms to influence neighboring organisms because they are small molecules that can easily diffuse through porous soils and travel over a long distance via the atmosphere. Ryu *et al.* [10] showed that certain bacterial VOCs function as signaling molecules for mediating plant-microbe interactions. They identified 2,3-butanediol and acetoin as VOCs that promoted the growth of *Arabidopsis thaliana*. Farag *et al.* [18], using SPME-GC-MS, identified an array of volatile compounds emitted from rhizobacteria that are involved in plant growth promotion. The capability of *Trichoderma* spp. to produce a great number of volatile secondary metabolites (e.g., pyrones, sesquiterpenes) has been reviewed [19]. Volatile secondary metabolites have been demonstrated to play a key role in mycoparasitism of *Trichoderma* as well as its interaction with plants [4].

**Figure 3.**
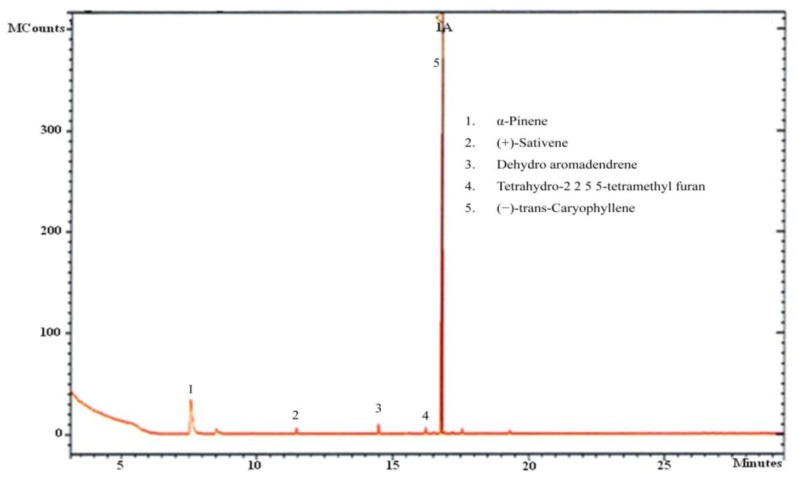
SPME GC-MS spectrum of volatiles of CL-1. (Volatiles present at undetectable levels/low quantities were not considered in this study).

**Figure 4.**
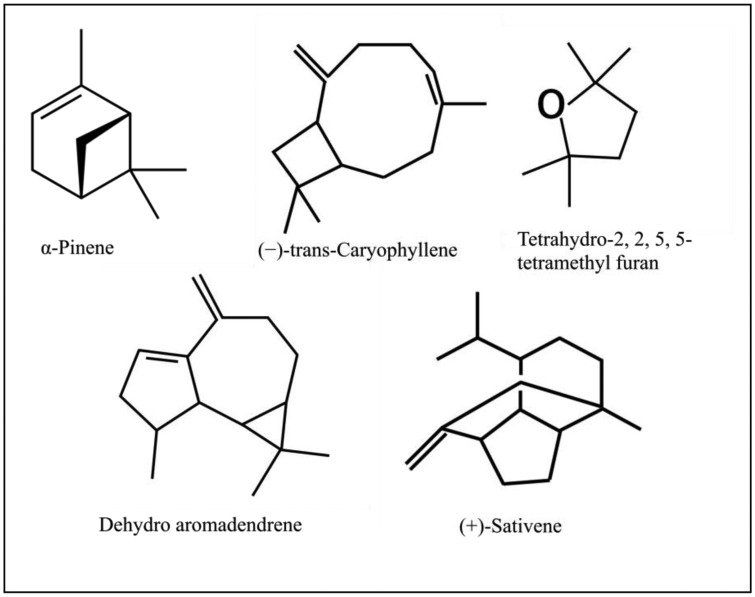
Volatiles from CL-1 by SPME/Quadrupole GC/MS/MS.

Our analysis of CL-1 volatiles using SPME/Quadrupole GC/MS/MS revealed that CL-1 emits the volatiles α-pinene, (−)-*trans*-caryophyllene, tetrahydro-2,2,5,5-tetramethylfuran, dehydro- aromadendrene, and (+)-sativene (Figures 3 and 4), which might have contributed to the enhanced seedling growth of tobacco. Monoterpenes including α-pinene are known to play important chemo-ecological roles in the interactions between plants and their environments, often playing a protective role against herbivores and pathogens [20]. Abraham *et al.* [21] reported that α-pinene at concentrations of 0.05–1.0 mM stimulated germination, primary root growth, and mitochondrial respiration of maize. Although at very high concentrations α-pinene has been reported to adversely affect plant growth and development [22]. The mechanisms by which monoterpenes affect seed germination are not known. The volatile sesquiterpene *trans*-caryophyllene is a major component in the essential oils of various medicinal plants, but its plant growth promoting effect has not yet been described. Yamagiwa *et al.* [11] reported the plant-growth-promoting fungus (PGPF), *Talaromyces* sp. emitted β-caryophyllene that significantly enhanced the growth of *Brassica campestris* seedlings and their resistance to *Colletotrichum higginsianum*. Zou *et al.* [23] showed that the VOC 2-pentylfuran, emitted by the PGPR *Bacillus megaterium* strain XTBG34, caused growth promotion in *A. thaliana*. Furan compounds have been reported to exhibit plant growth regulatory properties and are therefore recommended for commercial use in agriculture to improve and control plant health. Farag *et al.* [18] also showed the PGPRs *B. subtilis* GB03 and *B. amyloliquefaciens* IN937a emit the volatile 2-pentylfuran at very low levels. There are also reports of fungal (*Aspergillus fumigatus*, *Fusarium* spp., *Aspergillus terreus*, *Aspergillus flavus*, *Aspergillus niger*) emission of furans [24]. Furans and their derivates are a potentially important, but poorly studied, class of VOCs and their mechanism of plant growth promotion is not well understood. Studies on the role of the microbially produced volatiles dehydroaromadendrene and (+)-sativene on plant growth are also lacking.

The function and exact mechanisms of action of most microbial VOCs are poorly understood. However Zhang *et al.* [25] reported that *B. subtilis* GB03 volatiles regulate the homeostasis of auxin and cell expansion and augment photosynthetic capacity by increasing the photosynthetic efficiency and chlorophyll content. Although the molecular process underpinning plant growth promotion are not known, the ethylene and cytokinin signaling pathways appear to be involved [10]. Ryu *et al.*[10] also suggested that the synthesis of bioactive VOCs is a strain-specific phenomenon and the exogenous application of such VOCs result in a dose-dependent stimulation of plant growth.

Identification of microbial strains that release VOCs that promote plant growth is of much concern more recently. VOCs can permeate air-filled pores of soils and can travel long distances [26] and this property is an added advantage for the fungal biocontrol agents. The potential of certain fungal VOCs in ISR and also enhancing the biocontrol properties of bacterial biocontrol agents have been described [27]. Also fungal VOCs that have antagonistic properties against other microbial plant pathogens have been reported [28,29]. Studies using mutant strains that are defective in producing each of these VOCs identified in the current study will likely offer clues to the function of these compounds, and application of synthetic versions of these VOCs would confirm their role in plant growth promotion. Further research is warranted to understand whether these VOCs induce growth promotion singly or in combination.

## 4. Conclusions

The fungal plant growth promoting agent, *Cladosporium cladosporioides* CL-1 enhanced the growth of tobacco seedlings even when the seedlings were grown physically separated from the CL-1 culture in an airtight chamber. Identification of volatiles of CL-1 revealed mainly α-pinene, (−)-*trans*-caryophyllene, tetrahydro-2,2,5,5-tetramethylfuran, dehydroaromadendrene and (+)-sativene. Further studies using mutant strains and synthetic analogues of these MVOCs are warranted to confirm the role of these MVOCs in plant growth promotion. Identification of suitable strains that release MVOCs and evaluation of it for plant growth promotion would help to identify suitable MVOCs and the commercial production and application of it promises potential plant growth promotion in crops grown under greenhouse conditions.
